# The cost‐effectiveness of multi‐purpose HIV and pregnancy prevention technologies in South Africa

**DOI:** 10.1002/jia2.25064

**Published:** 2018-03-14

**Authors:** Matthew Quaife, Fern Terris‐Prestholt, Robyn Eakle, Maria A. Cabrera Escobar, Maggie Kilbourne‐Brook, Mercy Mvundura, Gesine Meyer‐Rath, Sinead Delany‐Moretlwe, Peter Vickerman

**Affiliations:** ^1^ Department of Global Health and Development London School of Hygiene and Tropical Medicine London UK; ^2^ Wits RHI University of the Witwatersrand Johannesburg South Africa; ^3^ PATH Seattle WA USA; ^4^ Center for Global Health and Development Boston University Boston MA USA; ^5^ Health Economics and Epidemiology Research Office Department of Internal Medicine Faculty of Health Sciences University of the Witwatersrand Johannesburg South Africa; ^6^ School of Social and Community Medicine University of Bristol Bristol UK

**Keywords:** discrete choice experiments, HIV prevention, key populations, multi‐purpose prevention, pre‐exposure prophylaxis, South Africa

## Abstract

**Introduction:**

A number of antiretroviral HIV prevention products are efficacious in preventing HIV infection. However, the sexual and reproductive health needs of many women extend beyond HIV prevention, and research is ongoing to develop multi‐purpose prevention technologies (MPTs) that offer dual HIV and pregnancy protection. We do not yet know if these products will be an efficient use of constrained health resources. In this paper, we estimate the cost‐effectiveness of combinations of candidate multi‐purpose prevention technologies (MPTs), in South Africa among general population women and female sex workers (FSWs).

**Methods:**

We combined a cost model with a static model of product impact based on incidence data in South Africa to estimate the cost‐effectiveness of five candidate co‐formulated or co‐provided MPTs: oral PrEP, intravaginal ring, injectable ARV, microbicide gel and SILCS diaphragm used in concert with gel. We accounted for the preferences of end‐users by predicting uptake using a discrete choice experiment (DCE). Product availability and protection were systematically varied in five potential rollout scenarios. The impact model estimated the number of infections averted through decreased incidence due to product use over one year. The comparator for each scenario was current levels of male condom use, while a health system perspective was used to estimate discounted lifetime treatment costs averted per HIV infection. Product benefit was estimated in disability‐adjusted life years (DALYs) averted. Benefits from contraception were incorporated through adjusting the uptake of these products based on the DCE and through estimating the costs averted from avoiding unwanted pregnancies. We explore the additional impact of STI protection through increased uptake in a sensitivity analysis.

**Results:**

At central incidence rates, all single‐ and multi‐purpose scenarios modelled were cost‐effective among FSWs and women aged 16–24, at a governmental willingness‐to‐pay threshold of $1175/DALY averted (range: $214–$810/DALY averted among non‐dominant scenarios), however, none were cost‐effective among women aged 25–49 (minimum $1706/DALY averted). The cost‐effectiveness of products improved with additional protection from pregnancy. Estimates were sensitive to variation in incidence assumptions, but robust to other parameters.

**Conclusions:**

To the best of our knowledge, this is the first study to estimate the cost‐effectiveness of a range of potential MPTs; suggesting that MPTs will be cost‐effective among higher incidence FSWs or young women, but not among lower incidence older women. More work is needed to make attractive MPTs available to potential users who could use them effectively.

## 1 Introduction

Over the past six years, clinical trials have shown that antiretroviral (ARV)‐based pre‐exposure prophylaxis (PrEP) can be efficacious in preventing the transmission of human immunodeficiency virus (HIV) 1, 2, 3, 4, 5. However, protection has been variable during clinical trials and different PrEP modalities have conferred substantially less protection to younger women than older women 4, 6. Both oral and topical PrEP products have been more effective in male populations than females 7, 8, partly explained by adherence, and partly by pharmacokinetic data indicating higher drug colorectal drug concentrations than in the female lower genital tract 6, 9.

It is likely that more than one effective prevention option will be needed to achieve population coverage among women 10. One option to increase impact while making products more desirable to potential users is to develop multi‐purpose prevention technologies (MPTs), which simultaneously provide protection from two or more of HIV, other STIs and unintended pregnancy 11, 12, 13, 14. Current MPTs in development include: (1) long‐acting drug delivery systems such as intravaginal rings, designed to protect from HIV infection and pregnancy (currently in phase‐1 trial, ClinicalTrials.gov Identifier: NCT02235662); (2) pericoital drug delivery systems such as vaginal gels, tablets and films (not currently being trialled); and (3) co‐provision of a combination of products, such as a contraceptive diaphragm with microbicide gel (currently feasible but not widely implemented) 11, 15. In the medium term, the co‐provision of contraceptive and HIV preventative products could be an intermediate step to the development of a MPT. As HIV prevention products come to market, provision alongside contraceptives – particularly when products are of the same modality – could facilitate uptake and adherence, and gauge demand for potential MPTs in the future.

Despite large variations in effectiveness and cost assumptions, previous studies have broadly found single‐purpose PrEP to be cost‐effective when delivered to populations at high risk of HIV infection 16, 17, 18, 19, 20. Although the financial and regulatory burden of MPT development is likely to be high, their benefits may also be large, not least because of synergistic impacts across health outcomes. Firstly, products offering more than one indication may be more attractive to some potential users than single‐purpose products, particularly the combination of contraceptive and HIV prevention properties 21, 22. Secondly, MPT use could *crowd‐in* protection from lesser valued attributes. For example, where users value contraceptive properties strongly, additional HIV protection would be a positive externality from the use of a dual‐protective product. In addition, we can use lessons learnt in contraceptive provision for different female populations to optimise MPT or co‐provision modalities 23. Thirdly, the contraceptive properties of MPTs may reduce the stigma associated with accessing HIV prevention tools, shown to be a substantive barrier to use 24, 25.

Unlike other PrEP impact models which use expert opinion to inform uptake assumptions, we take a data‐driven approach using a discrete choice experiment (DCE) to predict product uptake among different groups 26. DCEs are economic tools which ask people to choose between hypothetical alternatives, and have been shown reliable when predicting real‐world choices 27, 28. Although relying on hypothetical choices, a DCE is useful because preferences can be elicited for products which do not exist yet, thus we can estimate demand for co‐formulated or co‐provided products containing contraceptive properties. DCEs are objective, end‐user focused tools which may be less biased than predictions based on expert opinion 28, 29.

This paper presents a cost‐effectiveness analysis which estimates the incremental benefits and health system costs of single‐ and multi‐purpose prevention products, compared to current practice of condom use and male circumcision prevalence. Cost‐effectiveness is modelled across three female groups due to differences in epidemiology and HIV risk in each: younger women (aged 16–24), older women (aged 25–49) and female sex workers (FSWs).

## 2 Methods

### 2.1 Analytic overview

**Figure 1:**
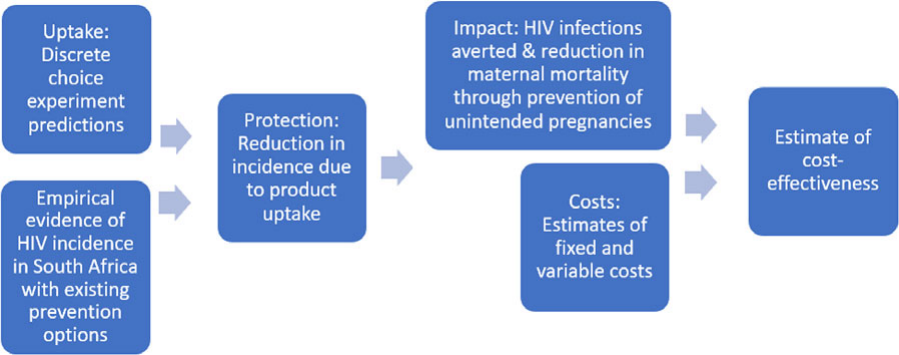
Modelling schematic.

This analysis builds on previous work using a DCE to elicit preferences for new HIV prevention products in South Africa 29. Figure 1 displays our approach to estimate the cost‐effectiveness of the five single and multi‐purpose prevention products considered: oral PrEP, intravaginal rings, injectable ARVs, microbicide gels and SILCS diaphragms used in concert with gel. A full list of parameters used in the model is presented in File S1. In this paper, we focus on contraceptive MPTs (considering STI prevention in a sensitivity analysis) because no STI‐specific MPT is in development while there are limited data on the efficacy of MPT products to prevent STIs. We model cost and benefits associated with one‐year's products use, and therefore do not consider variations over time such as increasing economies of scale or diminishing adherence patterns.

### 2.2 Estimating uptake

DCE data were gathered in Ekurhuleni Municipality, South Africa between September and December 2015 from 484 self‐reported HIV‐negative women: 362 from the general population and 122 FSWs. We use DCE data to simulate uptake for different scenarios of MPT characteristics (Table 1) in each population. In the model, users choose one product (or continue current practice) from a set, where availability is defined by different policy scenarios set out in the next section. The DCE protocol and results are described in detail elsewhere 26, 30, while further information is given in File S2.

**Table 1:** Product characteristics

Product characteristics	HIV efficacy (%, E_x_), [PSA bounds]	Contraceptive protection	Frequency of use	Product cost assumptions ($USD)	Source
Single‐purpose				Direct cost of single purpose ARV product	
Oral PrEP	61 [40–75]	N	Daily	75 [70–130] (/person‐year)	19, 31, 32
Microbicide gel	55 [31–71]	N	Coitally	3.69 [3–4.5] (/tube)^b^	Manufacturer (Kessel 33)
SILCS diaphragm	55 [31–71]^a^	N	Coitally	5.19 [4–6] (/diaphragm)	Manufacturer (Kessel 33)
Vaginal ring	55 [31–71]	N	Monthly	6 [5–7] (/ring)	Distributor (IPM 34)
Injectable ARV agent	75 [55–90]	N	Three monthly	6 [5–7] (/injection)	Assumption from vaginal ring
No condom	0	N	Coital	N/A (comparator)	
Multi‐purpose				Marginal direct cost of contraceptive compound	
Male condom	95 [66–94]	Y	Coital	N/A (comparator)	
MPT oral PrEP	61 [40–75]	Y	Daily	8.72 [6.17–11.5] (/person‐year)	35
MPT microbicide gel	55 [31–71]	Y	Coitally	9.14 [6.4–12] (/person‐year)	35
SILCS diaphragm & microbicide gel	55 [31–71]^a^	Y	Coitally	–	
MPT vaginal ring	55 [31–71]	Y	Monthly	9.14 [6.4–12] (/person‐year)	Assumed equal to highest cost product (injectable) 35
Injectable MPT agent	75 [55–90]	Y	Three monthly	9.14 [6.4–12] (/person‐year)	35

^a^Due to co‐use, the efficacy of the SILCS diaphragm was assumed the same as the microbicide gel; ^b^Assumptions on product use and other associated costs of provision listed fully in File S3.

### 2.3 Rollout scenarios

We modelled product uptake for five rollout scenarios that differed in product characteristics and availability. Table 2 shows the scenarios modelled, for all of which we use current practice as the base case for comparison. Scenario 1 is the most likely to occur first, as single‐purpose oral PrEP is already available to FSW groups in South Africa 36. Cautiously positive results from intravaginal ring trials 5 indicate that a single‐purpose ring with HIV prevention may be introduced next, while a multi‐purpose ring could be available in the medium term; we refer to this MPT ring plus PrEP scenario as scenario 3 11. Finally, we model the introduction of a varied suite of single then multi‐purpose products in scenarios 4 and 5 respectively. Although poor efficacy in clinical trials has seen the HIV prevention field move away from microbicide gels 8, we include them here to inform the development of other topical ARV‐based preventative methods, including vaginal tablets or films 37.

**Table 2:** Product scenarios modelled

	Product(s)	HIV protection	Pregnancy protection
Referencescenario	Current male condom usage. No ARV‐based single‐ or multi‐purpose prevention		
Scenario 1	Oral PrEP	X	
Scenario 2	Oral PrEP	X	
	Vaginal ring	X	
Scenario 3	Oral PrEP	X	
	MPT vaginal ring	X	X
Scenario 4	Oral PrEP	X	
	Intravaginal ring	X	
	Injectable ARV agent	X	
	Microbicide gel	X	
	SILCS diaphragm & microbicide gel	X	X
Scenario 5	MPT oral PrEP	X	X
	MPT vaginal ring	X	X
	Injectable MPT agent	X	X
	MPT Microbicide gel	X	X
	SILCS diaphragm & microbicide gel	X	X

### 2.4 Estimating HIV protection

To be conservative, and to focus on differences between populations and the relative preferences for different products, we do not model temporal reductions in the overall level of HIV transmission due to long‐ term product use. Instead, we model the yearly impact of introducing each product scenario among three female population groups in South Africa, and compare the total and incremental costs and benefits of each introduction scenario.

We use formula (1) to estimate the impact of each product scenario on the average level of protection conferred to an individual 29, 38. This measure gives the average decrease in HIV transmission probability across a population for an average sex act, combining the protection provided from several products with different efficacies being used at different levels. For a single product *x*, we assume the average protection against HIV, *p*_*x*_, from using product *x* is a function of its efficacy, *E*_*x*_, and uptake (or use) *U*_*x*_,

(1) $$p_{x}\;=\;E_{x}U_{x}$$

We begin by denoting *P*_*x*_ as the existing protection provided by male condoms with efficacy *E*_*0*_ and consistency of use *U*_*0*_, before any new products are introduced our DCE then gives projections of the degree to which condom users and non‐condom users (defined by use at last sex act) uptake each new product in each scenario *s*, and for condom users the probability that the woman still uses the condom (ε) in addition to the new products. For scenarios involving a number *m* of new products (denoted by subscript i = 1*…m*), each with efficacy $E_{i}^{s}$ and differing uptake $U_{\mathrm{ic}}^{s}$ among condom users (*c *=* *1) and non‐condom users (*c *= 0) respectively, the average protection provided to an individual in the population due to condoms and the introduction of *m* new products (not just among users of the product) is estimated as $P_{m}^{s}$:

(2) Pms=U0E0−∑i=1…mUi,1s(1−ε)E0+(1−ε)∑i=1…mEisUi,1s+ε∑i=1…m(1−E0)EisUi,1s+(1−U0)∑i=1…mEisUi,0s

The uptake of each product among condom and non‐condom users depends not only on the efficacy of the product for preventing HIV but also on whether they provide pregnancy protection. The prevention protection provided by different products is assumed to be additive. Users are assumed to use only one new ARV‐based prevention product, except the diaphragm and gel which are necessarily used together for MPT protection. When condoms are used with a new product, the new product was assumed to proportionately decrease the remaining risk still existing after the 85% protection provided by the condom 39. The uptake of each new product $U_{\mathrm{ic}}^{s}$ can be defined as uptake multiplied by adherence, and we vary the latter in a one‐way sensitivity analysis and both in probabilistic sensitivity analysis.

For oral PrEP, we assumed an HIV efficacy of 61% (40–75% used in probabilistic sensitivity analysis) as found in a recent meta‐analysis of the efficacy of oral PrEP among highly adherent women (>75% adherence) 40. We then assume that the vaginal ring is introduced with a HIV efficacy of 55% (31–71% used in probabilistic sensitivity analysis), as found among older women in a recent trial 41. Although some clinical trials of microbicide gels have shown a lack of efficacy, they have been shown to be efficacious in others and work continues to develop a gel which could also offer multi‐purpose protection 11. We assume that a microbicide gel would not be introduced if it was less effective than the next‐least effective product, in this case the vaginal ring, and we use the same uncertainty bounds and allow each to vary in probabilistic simulations. A higher efficacy for HIV protection of 75% (55–90% used in probabilistic sensitivity analysis) was assumed for injectable ARVs because there should be fewer issues of adherence.

### 2.5 Estimating DALYs averted through preventing HIV infection

Our simple impact model estimates the number of infections averted through one year's use of different product scenarios, where incremental protection, $P_{m}^{s}$, is used to decrease HIV incidence. We estimate the total discounted lifetime treatment costs averted from these infections. We use World Bank estimates of the size of the general population in South Africa in 2017 among two female groups aged 16–24 and 25–49 42 and apply HIV prevalence estimates from the South African HSRC National HIV Prevalence, Incidence and Behaviour Survey 2012 43 to estimate the HIV negative population in each group. For FSWs, we used national estimates of FSW population size and HIV prevalence in South Africa 44, 45. Standard DALY weights are used for HIV/AIDS with and without ART taken from the 2010 Global Burden of Disease study 46, full details of the DALY calculations are listed in Files S1 and S3.

We obtained group level incidence estimates from national surveys, as well as from recent HIV treatment and prevention trials in South Africa 40, 43, 47, 48 presented in Table 3. For each group, we model scenarios of low, central and high estimates of incidence. The level of protection provided by each scenario, $P_{m}^{s}$, is used to model a decrease in incidence:

(3) $${\text{Incidence}}_{\mathrm{new}}\;=\;{\text{Incidence}}_{\mathrm{current}}*\left(1-P_{m}^{s}\right)$$

**Table 3:** Estimates of HIV incidence per 100 person years

	Low incidence	Central incidence	High incidence	References
Females 16–24	1.62	2.54	5.00	41, 48, 49
Female 25–49	1.20	1.62	3.50	41, 48, 49
FSW^a^	3.50	5.00	8.00	8

^a^No FSW incidence data found, instead high level female incidence figures were used.

### 2.6 Estimating unintended pregnancies averted

We take the median projected unmet need for contraception among females in the general population as defined by the United Nations Population Division 50, and assume that FSWs who report using no method of contraception have unmet need. Informed by DCE estimates, in the initial comparison case we assume that 20% of women with unmet need will use products with contraceptive properties, and explore how variation in this parameter affects results in a sensitivity analysis. Following the family planning literature, DALYs are averted solely from the reduction in estimated maternal mortality from pregnancies averted through MPT use 51, 52.

### 2.7 Estimating costs

We estimate costs related to the delivery of a combination prevention package across all South African public clinics from a health system perspective. Included intervention costs are listed in Table 4, and details of their estimation given in File S3. Products have varying frequencies of collection and use which we model using realistic clinical use scenarios, informed by the South African national guidelines for PrEP rollout among high‐risk groups 36. Where MPT products do not yet exist, we used existing contraceptive product costs to account for costs of additional active compounds 35. Lifetime averted costs were estimated by multiplying the number of HIV infections and unwanted pregnancies averted by the discounted lifetime cost of ART treatment (using estimates of life expectancy on ART 53), and delivery, or abortion costs respectively.

**Table 4:** Included intervention costs

Fixed costs	National start‐up costs	Training of providers Mass media
Variable costs (based on predictions of use)	Facility distribution costs	Staff time Product Screening tests Health system mark‐up and overheads
	Averted health costs	Antiretroviral treatment Miscarriages and births from unplanned pregnancies

### 2.8 Sensitivity analyses

We present results for each group and each scenario at high, medium and low incidence estimates. In addition, we perform a series of one‐way deterministic sensitivity analyses to explore how changes in qualitatively important characteristics affect estimates. Finally, a probabilistic sensitivity analysis (PSA) explored the sensitivity of our model to a range of parameter uncertainties, using a Monte Carlo simulation with 1000 draws. Point estimate and distributional assumptions for the variables included in the PSA are listed in File S1, and results presented in File S8.

### 2.9 Assessment of cost‐effectiveness

We assessed the cost‐effectiveness of different scenarios by computing the incremental cost‐effectiveness ratio (ICER) for each intervention: the net costs of an intervention divided by the number of DALYs averted, and compare this to a willingness‐to‐pay (WTP) threshold. The South African government does not have a generally accepted WTP threshold; in the absence of this we use the threshold estimates of Woods *et al*. 54 to represent the opportunity cost of health forgone from other potential interventions. We took a conservative stance and applied the lowest estimate, the lower‐bound USD threshold of $1175/DALY averted (range: $1175–$4714), which was lower than the purchasing‐power adjusted range ($2221–$8909) and alternative thresholds such as 1× and 3× GDP/capita 54, 55.

## 3 Results

### 3.1 Deterministic model analysis

**Figure 2:**
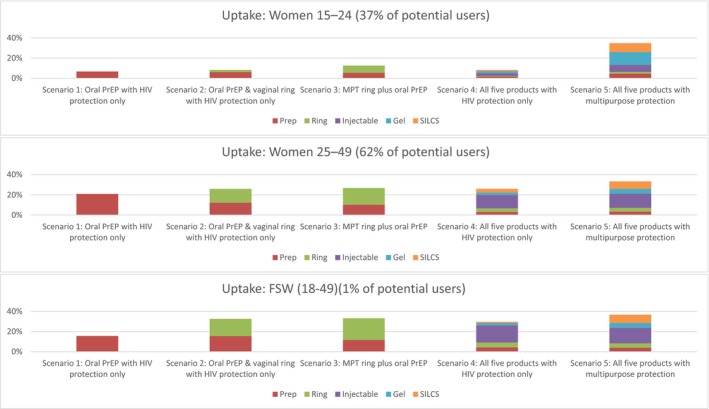
Product uptake by group.

Results suggest that co‐provision or co‐formulation of contraceptive and HIV prevention products will be cost‐effective among younger women and FSWs, but not among older women. Younger women show distinct preferences for multi‐purpose over single‐purpose products as shown in Figure 2 which aggregates uptake among condom and non‐condom users. Notably, we predict that just 8% of women aged 16–24 would use at least one of the full range of single‐purpose HIV prevention products, however uptake increases by 27 percentage points if products were co‐formulated as MPTs. The predicted uptake of single‐purpose products is much greater among older women (26%) and FSWs (30%), but additional uptake from including pregnancy protection is relatively low at 7 and 6 percentage points respectively. Where all products are introduced, the decrease in HIV incidence attributable to additional uptake due to multi‐purpose protection was higher among younger women (reduction in 19%) than among other groups (8% for older women and FSWs).

**Figure 3:**
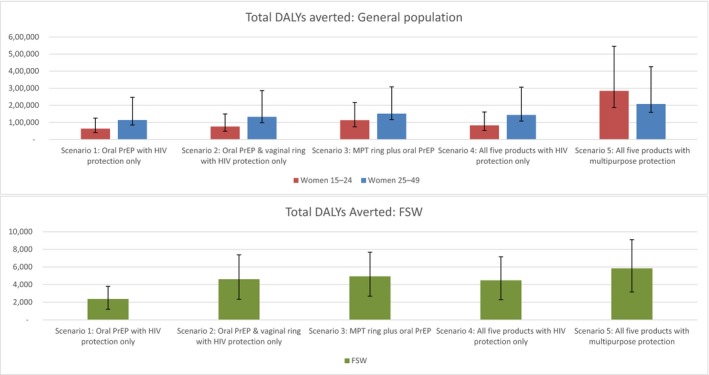
DALYs averted by population, scenario, and incidence assumption. Coloured bars represent DALYS averted at central incidence estimates, with upper and lower incidence assumptions indicated for each scenario. Populations presented separately due to scale differences in small FSW population.

Of the 12.9 million women aged 15–49 in 2017, 10.1 million were estimated to be HIV negative 43. Assuming central HIV incidence estimates, 201,552 new HIV infections are predicted to occur without intervention. Figure 3 illustrates DALYs averted for each product scenario among each population group. We focus here on cost‐effectiveness, but present full impact results in File S6. Net intervention costs are presented in Figure S7, where rollout among the 1.7‐times larger population of women aged 25–49 leads to a four‐fold increase in net intervention costs than among those aged 16–24.

**Figure 4:**
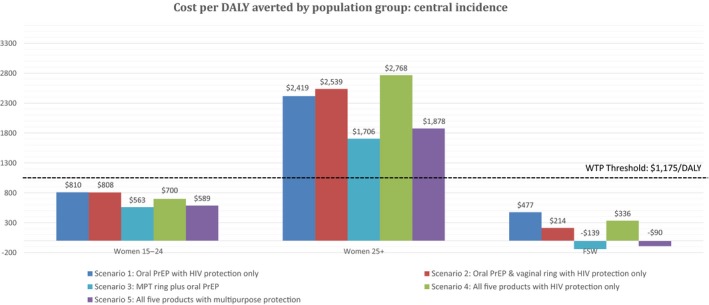
Average cost per DALY averted by scenario and incidence assumption. Nb. Negative ICERs among FSWs are cost‐saving and DALY increasing interventions with a positive impact.

Figure 4 shows ICERs for each product scenario and population group. For all groups, oral PrEP plus a MPT intravaginal ring (Scenario 3) is the most cost‐effective scenario modelled, with an ICER of $563/DALY averted among younger women and dominating the comparator among FSWs. All scenarios modelled among women aged 16–24 and among FSWs are cost‐effective when compared to the WTP threshold of $1175, whilst the no rollout status quo scenario is cost‐effective among women aged 25–49. Among all populations, scenarios where multi‐purpose products are introduced are more cost‐effective than any combination of single‐purpose products.

These results show the cost‐effectiveness of interventions relative to current levels of condom use, yet the increasing availability of oral PrEP in South Africa mean that a more meaningful comparator may be that of oral PrEP provision. Table 5 displays ICER estimates for introducing single‐ and multi‐purpose products, with the comparator of oral PrEP and the range of single‐purpose products that may be available. Again, results indicate that at under all incidence assumptions, introducing additional ARV‐based prevention products will be cost‐effective among women aged 16–24 and FSWs. Results differ among women aged 25–49, where introducing additional MPT products is estimated to be cost‐effective (in most cases cost‐saving), but adding a single‐purpose ring to provision of single‐purpose PrEP is not cost‐effective except under assumptions of high incidence.

**Table 5:** ICER values of comparative scenarios

Incidence assumption	Women 16–24			Women 25–49			FSW		
	Low	Central	High	Low	Central	High	Low	Central	High
Single‐purpose									
Adding vaginal ring to PrEP (Scenario 2 compared to Scenario 1)	$1691	$801	$30	$4763	$3282	$1008	$792	$‐66^a^	$‐398^a^
Multi‐purpose									
Adding MPT ring to PrEP (Scenario 3 compared to Scenario 1)	$727	$243	$‐225^a^	$‐401^a^	$‐482^a^	$‐669^a^	$‐532^a^	$‐709^a^	$‐791^a^
Adding MPT range to single‐purpose range (Scenario 5 compared to Scenario 4)	$1214	$543	$‐79^a^	$88	$‐114^a^	$‐503^a^	$‐1810^a^	$‐1502^a^	$‐1334^a^

^a^Negative ICER values indicate cost‐saving interventions with a positive impact.

### 3.2 Sensitivity analyses

**Figure 5:**
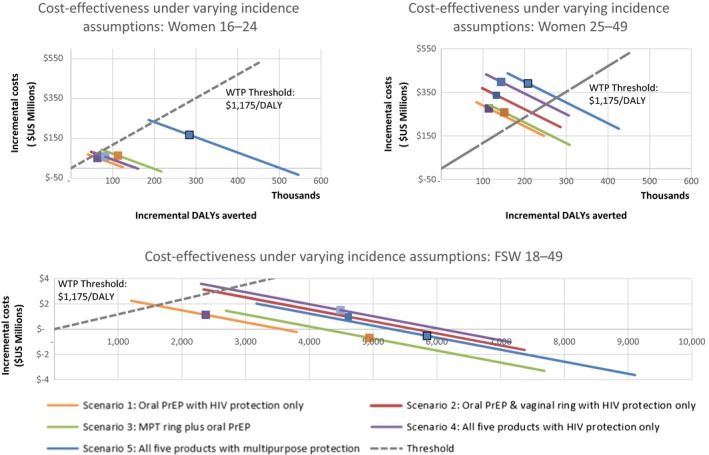
One‐way sensitivity analysis on incidence assumptions.

Figure 5 shows one‐way sensitivity analyses for incidence assumptions. Our base case model assumptions are represented by squares, whilst lines to the upper‐left and lower‐right demonstrate ICER estimates at lower and upper incidence assumptions respectively. Points to the south‐east of the dashed WTP line are interpreted as cost‐effective. In all but two scenarios modelled (both among FSWs), using an upper or lower incidence assumption means that the estimated ICER crosses the willingness‐to‐pay threshold. The incidence level at which the most cost‐effective scenario became cost‐ineffective was 1.3, 1.6 and 1.1 infections per 100 person‐years for women aged 16–24, 25–49 and FSWs respectively.

**Figure 6:**
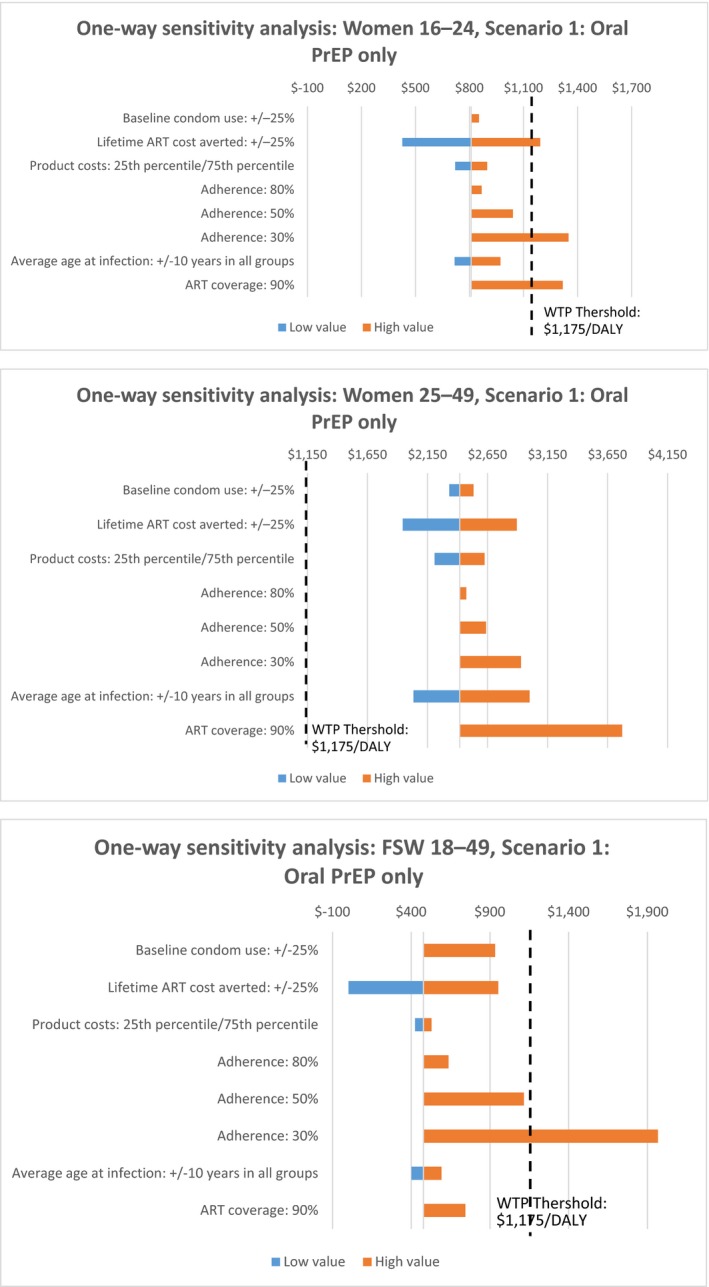
One‐way sensitivity analyses of scenario 1.

Further one‐way sensitivity analyses explored the effect of parameter uncertainty on model outputs. Tornado plots for scenario 1 are presented in Figure 6 which became cost‐ineffective among women aged 16–24 and FSWs when adherence was assumed to be 30%, or if ART coverage was increased to meet the 90% WHO target 56. Including STI preventative attributes in addition to HIV and pregnancy does not markedly change cost‐effectiveness estimates. Tornado plots for the most cost‐effective scenario (oral PrEP plus MPT ring) are included in File S6, and demonstrate cost‐effectiveness in females aged 16–24 and FSWs for all parameter variations.

MPT cost‐effectiveness was broadly robust to reductions in the assumed efficacy of products. Additional one‐way sensitivity analyses (not shown in Figure 6 were carried out on assumptions of product efficacy. Reducing the effectiveness of all products to 50% meant that all three scenarios of solely single‐purpose products would not be cost‐effective among women aged 16–49 (Scenario 1 had the highest ICER of $1281), though all MPT scenarios and scenarios among FSWs would remain cost‐effective. MPT scenarios among younger women were estimated not to be cost‐effective at an efficacy of 45%.

**Figure 7:**
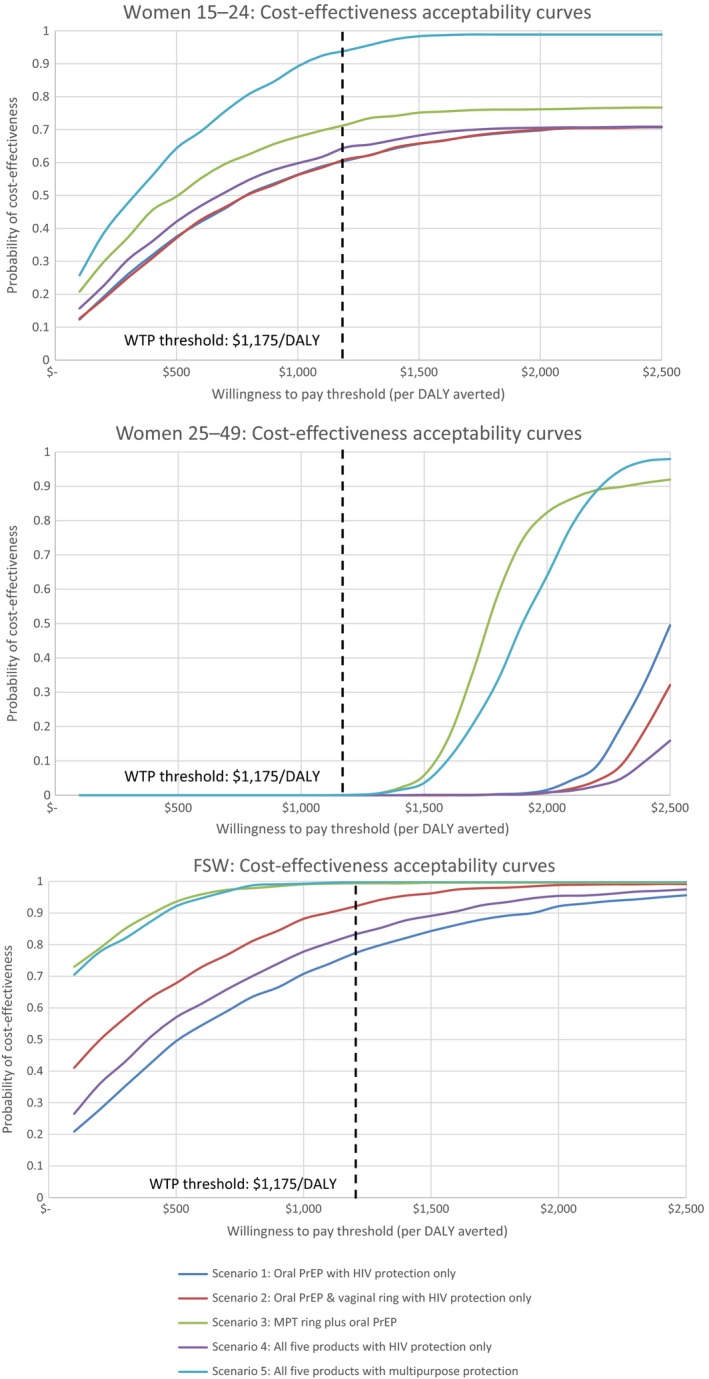
Cost‐effectiveness acceptability curves.

Figure 7 presents cost‐effectiveness acceptability curves for each scenario among each population, while simulation plots on the cost‐effectiveness plane are included in File S7. For all groups, both scenarios including MPTs were most likely to be cost‐effective. For older women, younger women, and FSWs respectively, the model predicts a 0.1%, 71% and 99% probability that the most cost‐effective scenario, scenario 3, will be cost‐effective. The PSA also confirms the primacy of interventions among FSWs and women aged 16–24, as even the least cost‐effective scenario for each group was 60% likely to be cost‐effective at the $1175 threshold.

## 4 Discussion

This study is the first to estimate the cost‐effectiveness of a range of candidate MPT products, and suggests that co‐formulated or co‐provided MPTs could be an impactful and efficient use of resources. Based on the stated preferences in the DCE, incorporating contraceptive characteristics into HIV prevention products would result in a meaningful increase in product use, reinforcing evidence of unmet demand for MPTs among many groups 11, 12. Results indicate that multi‐purpose prevention products are likely to be cost‐effective among younger women (aged 16–24) and FSWs compared to current condom provision, with scenario 3, oral PrEP plus a MPT ring, and scenario 5, the full range of MPT products, estimated to be the most cost‐effective. However, despite being cost‐effective, our uptake projections suggest that products are unlikely to achieve dramatic decreases in HIV incidence. Even if all MPT products were introduced, incidence among older women and FSWs is projected to reduce by just 8% (19% among younger women), far under the estimated 48% reduction from achieving UNAIDS’ 90‐90‐90 target 57. Although MPTs offer a cost‐effective option for tacking the HIV epidemic, a range of programmes will be required to reduce incidence substantively.

The co‐formulation or co‐provision of products is likely to increase programme cost‐effectiveness for two reasons. First, the multi‐purpose nature of the products makes them more attractive to potential users, increasing uptake and therefore economies of scale from product use. Second, the costs associated with unwanted pregnancies averted reduce the net costs of the intervention overall, and increase benefits accrued from averted maternal mortality. Incorporating STI prevention does not markedly change cost‐effectiveness estimates.

These estimates of cost‐effectiveness are broadly comparable to published studies, though there is considerable heterogeneity in these 20. Although we base our counterfactual scenario on empirical data from trials and nationally representative population based studies, our simple incidence model predicts a higher number of annual infections than more complex models, such as Thembisa and EPP/Spectrum models 58, 59. The reason for this is uncertain, although it is worth noting that the both models underestimate the 2012 HIV incidence in South Africa compared to empirical estimates by around 0.4 infections/100 person years among women aged 16–29 60. In addition, when incidence is varied in our sensitivity analysis, our results are consistent with other models. We note that the relatively low effectiveness figures observed in topical PrEP trials (e.g. 5, 61) may reduce the likelihood of governments investing in them, but incorporating additional benefits such as contraception into partially effective products may increase their chances of introduction.

This study has several limitations. First, we used a simple static transmission model to estimate the short‐term benefits of introducing different products. This model does not consider prevention benefits accruing into the future, including the dynamic effect of reductions in incidence on prevalence. However, this simplicity is also a strength because it gives transparent estimations of the individual benefits of using these products, resulting in estimates of impact and cost‐effectiveness similar to what would be produced from a trial. Unfortunately, although dynamic transmission models can be more realistic in capturing the longer‐term benefits of an intervention, their reliance on numerous assumptions, particularly around the future dynamics of infection, makes their projections uncertain. Indeed, a recent analysis of 12 models for South Africa showed that none were able to correctly predict the future dynamics of infection between 2006 and 2011 62. Importantly, our use of a static model results in conservative impact projections, as a dynamic model would predict increased numbers of secondary infections averted with product use. However, when new preventive methods are used by a small proportion of the population, as predicted here, total population protection has been shown similar to a static model of efficacy multiplied by use 63, suggesting our model projections may be fairly accurate.

Second, the model does not incorporate differential adherence between products, although the one‐way sensitivity analysis shows that adherence could be less than 50% and products still be cost‐effective. Third, results are sensitive to incidence assumptions, and 8 of the 10 cost‐effective scenarios become cost‐ineffective if low HIV incidence assumptions are used. However, a PSA shows that MPT scenarios among FSWs and women aged 16–25 are 99% and 71% likely to be cost‐effective at the conservative $1,175 threshold respectively. Fourth, we do not consider reductions in paediatric HIV incidence due to unintended pregnancies averted, making estimates conservative. Fifth, we consider one‐year estimates of cost and benefit from each scenario. In reality, this may overestimate costs due to the potential for economies of scale in delivery over time. Estimates of benefit could also be overestimated, if uptake is graduated over time leading to the level assumed here, or if adherence was assumed to vary over time, reducing as a function of length of use for example. Sixth, we estimate the costs of multi‐purpose products to be additive of that to single‐purpose products, which may underestimate the true cost of products, particularly if co‐formulated, in a real‐world value‐based pricing framework. This means that our cost estimates for MPTs may be too low, making MPTs seem more cost‐effective than they may be in reality, however since the estimated costs of oral PrEP are substantively higher than those observed in real‐world demonstration projects (e.g. 64), the overall impact may be mitigated. Finally, although single‐purpose contraceptives are available for all delivery mechanisms modelled (oral, injectable and topical products), there is variation in which mechanisms are being developed for co‐formulation to provide multi‐purpose prevention. The introduction of a range of five MPTs is unlikely to occur in reality.

This paper is also the first to use a discrete choice experiment to predict product uptake within a cost‐effectiveness analysis in HIV, which allows us to explicitly consider heterogeneity in end‐user preferences between different groups. While DCE survey data were randomly sampled and reweighted to match the age structure of the female South African population, these data are unlikely to be generalizable to the entire country or other countries. Furthermore, the FSW data were collected through respondent driven sampling (RDS) and, although reweighted, the highly variable nature of sex work in South Africa makes reliable statements about generalisation difficult 45.

## 5 Conclusions

This study estimates the cost‐effectiveness of five candidate MPTs, and finds that co‐formulation or co‐provision of contraceptive and HIV prevention products would be an efficient and effective use of resources among younger female groups and FSWs at current levels of HIV incidence. Younger women in particular find MPTs more attractive than single‐purpose technologies, suggesting that incorporating contraceptive properties in HIV prevention products – or vice‐versa – could lead to substantive HIV and family planning benefits in this group. These results strengthen calls for more research and development in the co‐formulation or co‐provision of products to reduce unmet need in sexual and reproductive health services.

## Competing interests

The authors have no competing interests to declare.

## Funding

Fieldwork was supported by the Bill and Melinda Gates Foundation. MQ receives an Economic and Social Research Council 1 + 3 studentship. Support for the analysis of this project is made possible by the generous support of the American people through the United States Agency for International Development (USAID) under the terms of the HealthTech V Cooperative Agreement #AID‐OAA‐A‐11‐00051. The contents are the responsibility of LSHTM and PATH and do not necessarily reflect the views of USAID or the US Government. This study was supported by the STRIVE research programme consortium funded by UKaid from the Department for International Development. However, the views expressed do not necessarily reflect the department's official policies.

## Authors’ contributions

MQ, FTP, RE, MC, SDM and PV conceived, designed and tested the DCE. MQ, FTP and PV analysed the data and developed the cost‐effectiveness model. MQ, FTP, RE, MC, SDM, MM, MKB, GMR and PV wrote and revised the manuscript.

## Supporting information


**File S1** List of model parameters: cost‐effectiveness model.
**File S2** Information on DCE and the impact model.
**File S3** The cost model.
**File S4** Division of fixed costs across populations.
**File S5** HIV infections averted.
**File S6** One‐way sensitivity analyses.
**File S7** Net intervention costs.
**File S8** Additional data from probabilistic sensitivity analysis.
