# Supplementary material for: The cost‐effectiveness of multi‐purpose HIV and pregnancy prevention technologies in South Africa
Source: J Int AIDS Soc. 2018 Mar 14;21(3):e25064. doi: 10.1002/jia2.25064 (PMC5851344; doi:10.1002/jia2.25064)
Supplement: Supplementary file 1 — File S1 List of model parameters: cost‐effectiveness model. File S2 Information on DCE and the impact model. File S3 The cost model. File S4 Division of fixed costs across populations. File S5 HIV infections averted. File S6 One‐way sensitivity analyses. File S7 Net intervention costs. File S8 Additional data from probabilistic sensitivity analysis. [file JIA2-21-e25064-s001.docx]

**Supplementary Material for:**

The cost-effectiveness of combined HIV and pregnancy protection: An economic evaluation of multipurpose prevention products in South Africa

**Supplementary file S1: List of model parameters: cost-effectiveness model**

| Parameter | Point estimate | Lower Credible Interval/95%CI | Upper Credible Interval/95%CI | PSA Distribution (SE where included) | Source(s) |
| --- | --- | --- | --- | --- | --- |
| **Cost inputs** |  |  |  |  |  |
| **Product variable costs (per person year)** |  |  |  |  |  |
| Oral Prep | $289 | $269 | $309 | Uniform* | See supplementary material S3 |
| Vaginal Ring | $258 | $243 | $273 | Uniform* | See supplementary material S3 |
| Injectable | $212 | $192 | $230 | Uniform* | See supplementary material S3 |
| Microbicide Gel | $438 | $349 | $516 | Uniform* | See supplementary material S3 |
| SILCS Diaphragm and Microbicide Gel | $217 | $199 | $235 | Uniform* | See supplementary material S3 |
| **Product fixed costs** |  |  |  |  |  |
| Staff training across all facilities | $34,696,831 |  |  |  | Conservative assumptions using staff salary costs. 3 days, 100 staff each training day from four facilities (25 per facility), 5 nurse facilitators |
| Mass media (annual) | $5,000,000 |  |  |  | ([1](#_ENREF_1)) |
| Outreach cost | $403,920 |  |  |  | Assumption based on FSW sample size, one clinic with 12 outreach staff per 1,500 FSWs |
| **Population sizes** |  |  |  |  |  |
| Females 15-24 | 4,810,897 |  |  |  | ([2](#_ENREF_2)) |
| Female 25-49 | 8,156,317 |  |  |  | ([2](#_ENREF_2)) |
| FSW | 153,000 | 132,000 | 182,000 | Uniform | ([3](#_ENREF_3)) |
| **HIV Prevalence** |  |  |  |  |  |
| Females 15-24 | 11% | 10% | 13% | Normal (0.009) | ([4](#_ENREF_4)) |
| Female 25-49 | 29% | 27% | 32% | Normal (0.014) | ([4](#_ENREF_4)) |
| FSW | 74% | 59% | 83% | Normal (0.049) | ([4](#_ENREF_4)) |
| **HIV Incidence**** |  |  |  |  |  |
| Females 15-24 | 2.54 | 1.62 | 5.00 | Uniform | ([5-7](#_ENREF_5)) |
| Female 25-49 | 1.62 | 1.62 | 3.50 | Uniform | ([5-7](#_ENREF_5)) |
| FSW | 5.00 | 2.54 | 8.00 | Uniform | ([8](#_ENREF_8)) |
| **Contraception parameters** |  |  |  |  |  |
| Percentage of women married/in union (relationship) from data | 86% | 82% | 90% | Beta (0.022) | Primary data |
| Percentage of women in relationship with unmet contraceptive need | 72% | 66% | 78% | Beta (0.030) | Primary data |
| Percentage of FSWs with unmen contraceptive need | 16% | 11% | 21% | Beta (0.026) | Primary data |
| Probability of getting pregnant using product (year) (perfect use) | 14% | 18% | 14% |  | SILCS, winner NEJM ([9](#_ENREF_9), [10](#_ENREF_10)) |
| Likelihood of becoming pregnant with no product (/year) | 40% |  |  |  | ([11](#_ENREF_11)) |
| ART costs (year) | 4,586.99 |  |  |  | ([12](#_ENREF_12)) |
| **Parameters for DALY calculations** |  |  |  |  |  |
| Discount rate | 0.03 | 3% | 7% |  |  |
| Age weighting modulation factor | 1 |  |  |  | ([13](#_ENREF_13)) |
| Constant | 0.1658 |  |  |  | ([13](#_ENREF_13)) |
| parameter from the age weighting function | 0.04 |  |  |  | ([13](#_ENREF_13)) |
| DALY Weight: pre-ART | 0.051 |  |  |  | ([14](#_ENREF_14)) |
| DALY Weight: symptomatic, not linked to care | 0.221 |  |  |  | ([14](#_ENREF_14)) |
| DALY Weight: on ART | 0.053 |  |  |  | ([14](#_ENREF_14)) |
| DALY Weight: AIDS | 0.547 |  |  |  | ([14](#_ENREF_14)) |
| DALY Weight: Infertility | 0.006 |  |  |  | ([14](#_ENREF_14)) |
| ART Access rate | 0.72 |  |  |  | ([15](#_ENREF_15)) |
| **Product Efficacy** |  |  |  |  |  |
| Oral Prep | 61% | 40% | 75% | Uniform | ([16](#_ENREF_16)) |
| Vaginal Ring | 56% | 31% | 71% | Uniform | ([6](#_ENREF_6)) |
| Injectable | 75% | 55% | 90% | Uniform | Assumption based on lower adherence requirement, large uncertainty bound modelled |
| Microbicide Gel | 85% | 66% | 94% | Uniform | Assumption that a microbicide gel would not be introduced if it was less effective than the next-least effective product (vaginal ring) |
| SILCS Diaphragm and Microbicide Gel | 56% | 31% | 71% | Uniform | Assumption that a microbicide gel would not be introduced if it was less effective than the next-least effective product (vaginal ring) |

* Separate PSA was carried out on cost-model inputs as described in supplementary file S3. A uniform distribution used here so as to not prescribe a functional form on the results of this PSA, and to allow greater uncertainty around these estimates into the final model

** Uniform distribution used on incidence assumptions to avoid prescribing a specific functional form to these data, largely obtained from trials

**Supplementary file S2: Information on DCE and the impact model**

DCE data were gathered in October to December 2015 in Ekurhuleni Metropolitan Municipality through a randomised household survey among 158 adult females and 204 adolescent girls (aged 16-17), who self-reported as HIV negative, and data were reweighted by two age strata to the general population structure of South African women([18](#_ENREF_18)). A respondent driven sampling process collected data for 122 FSWs from the same geographical area, which was reweighted using RDSAT software([19](#_ENREF_19), [20](#_ENREF_20)).

**Figure S2:1: Example DCE task presented to participants:**


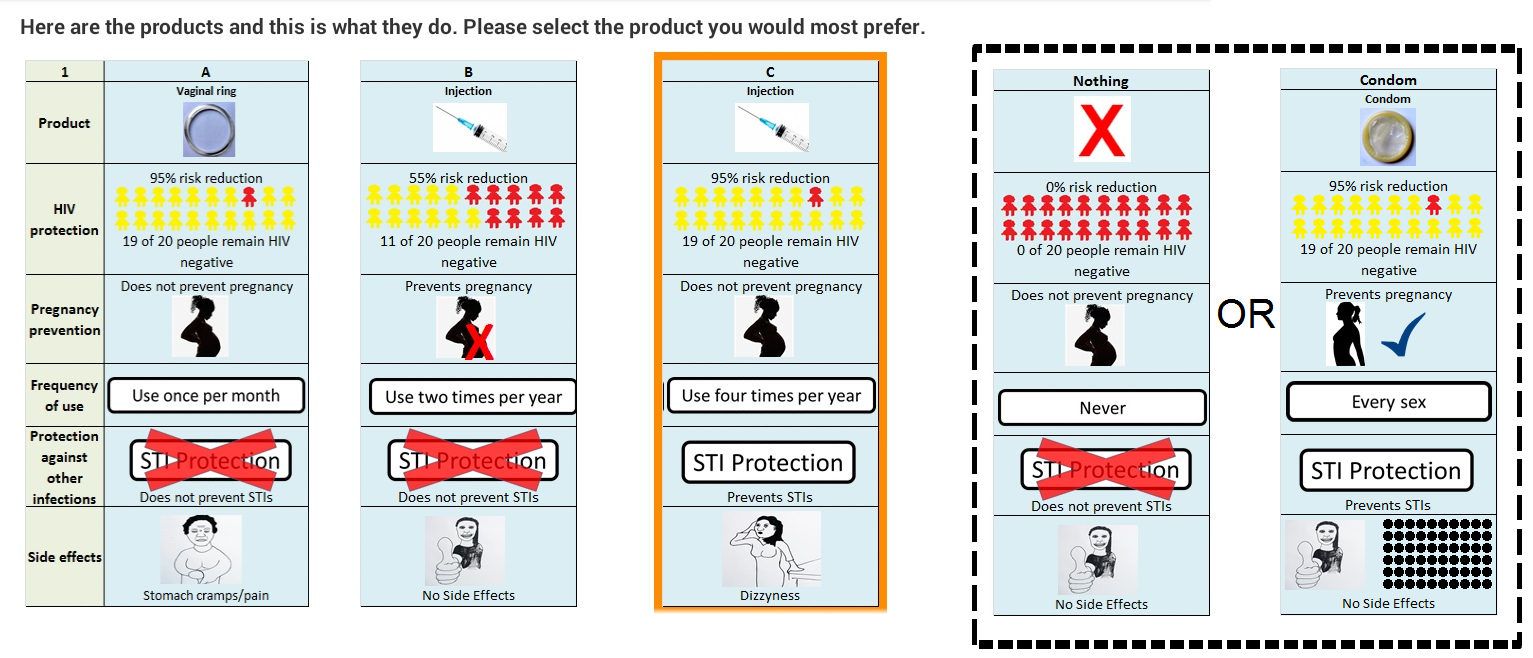


DCE data are used to predict uptake through predicted probability analysis, as described in earlier work.([21](#_ENREF_21)) Briefly, coefficients from DCE nested logit utility functions are summed according to expected characteristics of potential prevention products as shown in table 1 such that:

$$P_{n,i}=\frac{e^{V_{n,i}}}{\sum_{j=1}^{J} e^{V_{n,i}}}$$

DCE analysis is based on the assumption that people make rational choices to maximise utility.([22](#_ENREF_22)) By analysing what respondents choose in ten hypothetical choice sets presented to them, we are able to compute the probability of a respondent making a specific product choice depending on the characteristics of that product (based on real-world attributes of products), and defining what other products are available and their characteristics.([21](#_ENREF_21)) This method of simulating from choice data has been termed predicted probability analysis (PPA), and has been applied in fields of health, environmental and transport economics.([23](#_ENREF_23), [24](#_ENREF_24)) Although the DCE presented MPT options as co-formulations, these results could also inform the co-provision of contraceptive and HIV preventative products.

We estimate uptake among condom users and non-condom users for each scenario and among each population, and use data from the primary survey to assume that condom use in partnership with new products is 60%. In the probabilistic sensitivity analysis, variation in DCE coefficients (and therefore uptake) is simulated through drawing from a normal distribution using the mean and standard error reported by the discrete choice model. All DCE coefficients are varied in this manner.

**Impact model**

Formula 1 firstly takes the original protection provided by condoms ($U_{0}E_{0}$), removes the loss in protection due to new product users stopping using condoms ($U_{0}\sum_{i=1..m} U_{i,1}^{s}{\left( 1-\varepsilon\right)E}_{0})$, then adds the new product protection amongst those individuals that stop using condoms ($U_{0}\left( 1-\varepsilon\right)\sum_{i=1..m} {E_{i}^{s}U}_{i,1}^{s}$), the new protection amongst new product users that carry on using condoms ($U_{0}\varepsilon\sum_{i=1..m} {{\left( 1-E_{0} \right)E}_{i}^{s}U}_{i,1}^{s}$), and the added protection of individuals that did not use condoms before but now use the new products ($\left( 1-U_{0} \right){\sum_{i=1..m} {E_{i}^{s}U}_{i,0}^{s}}$).

**Supplementary file S3: The cost model**

**Cost model methods**

This cost model estimates the costs associated with the roll-out of five new antiretroviral(ARV)-based HIV prevention products: oral pre-exposure prophylaxis (PrEP), a microbicide gel, a SILCS diaphragm used in concert with microbicide gel, an intravaginal ring (IVR), and an injectable ARV. The main cost model takes a health system (provider) perspective. It considers the direct medical costs of provision for each method, associated health system costs, and costs averted from HIV infection alongside unwanted or mistimed pregnancies.

As per these guidelines, we assume that when first prescribed a prevention product, users will be tested for HIV infection immediately and after one month, then returning for a test every third month. We assume that all users receive the services detailed in the guidelines.

We carried out a probabilistic sensitivity analysis which explored the role of parameter uncertainty in the model. Inputs were obtained from published literature or computed from a primary survey carried out in Gauteng Province in late 2015 (survey protocol in appendix II) and from the 2003 DHS dataset.([11](#_ENREF_11), [25](#_ENREF_25)) Where data were unavailable through these sources, we contacted international experts and product-development sources for remaining parameters.

**The setting and distribution scenarios**

The model represented all South African public health clinics, a large network of 3,182 facilities, as the South African PrEP guidelines indicate that provision will be fully integrated within primary healthcare clinics to mitigate stigmatisation when trying to obtain PrEP.([26](#_ENREF_26)) We model that the introduction of the new products is via public facilities, supported by a mass media campaign run annually: for six months prior to rollout (at double intensity – and thus cost than that which would follow in later years. The model allows for differential demand on staff time by product, however do not include substantial variation across products in the base case in table 2. Different products have different frequencies of collection and use which we model using realistic clinical use scenarios informed by the South African national guidelines for PrEP rollout among high risk groups which requires persons using PrEP to obtain an HIV, and associated other medical, tests every three months.([26](#_ENREF_26)) As per these guidelines, we assume that when first prescribed a prevention product, users will be tested for HIV infection immediately and after one month, then returning for a test every third month. Finally, as per these guidelines, users will receive the tests detailed in table 3 before initiation, and during maintenance on products. We assume that all users receive the services detailed in the guidelines.

Whilst some products have a well-defined regimen, the SILCS diaphragm and microbicide gel are used coitally and oral PrEP daily, the newer products (namely the IVR and injectable ARV) are still in development and regimens as yet unknown. We use expert opinion to parameterise use for these products, defining both as products which require monthly application via a health facility. In a sensitivity analysis, we reduce the health system contacts required for efficacious use of these products.

**Distribution Costs**

The main cost model takes a health system (provider) perspective and presents the incremental costs of each new prevention product (total costs less costs averted by the introduction of the products). Table S3:4 (at the end of this supplementary file) displays model parameters and sources. Included are fixed provider costs, mass-media costs (at a country level) alongside with initial and refresher training (annually at a third of the initial intensity) at a provider level. [(1](#_ENREF_10)0) [(1](#_ENREF_10)0) [(](#_ENREF_9)9) [(](#_ENREF_7)7)([20](#_ENREF_20))([18](#_ENREF_18))([17](#_ENREF_17)) Provider training costs were based on personal communication with the female sex worker PrEP rollout in South Africa and based on actual training provision for the rollout of oral PrEP, as per Table S3:1. Training was held over three days, with around 25 participants from a facility per day comprising of 20 nursing staff and 5 HIV counsellors. Variable costs vary with the number of users and include direct product and associated testing costs, and user counselling costs. In addition to this, following the literature ([10](#_ENREF_10), [27](#_ENREF_27)), overhead costs are accounted for through the use of a facility mark-up factor which captures direct health facility costs as twice labour costs, alongside a health system cost (half of the facility mark-up) which captures resources spent on upper management and logistics.

It is anticipated that the initial introduction of products would incur large fixed costs relating to training and mass media, whilst later products would not need such a large volume of initial resources. So that the sequence of product introduction does not affect cost-effectiveness estimates, as presented in supplementary material S1, we assume a pool of common, fixed training ($34.7m) and mass media ($5m) costs before the introduction of any product. Because we assume that all products in a scenario are brought to market at the same time, fixed costs are divided equally across products introduced in each scenario. When estimating population level costs, the division of fixed costs is weighted proportionately to the number of users in each group, which is described fully in supplementary table S4. To estimate costs for MPTs that do not yet exist, we use cost estimates for presently available injectable and oral contraceptive products to estimate unit costs of compounds and associated health services.([28](#_ENREF_28)) These costs would be present if MPTs were either co-formulated or co-provided.

The SILCS diaphragm is assumed to have a 1 year useable life (likely a conservative estimate ([10](#_ENREF_10))), whilst gel, oral PrEP and IVR supplies are refreshed after a three month period when a user reports to a facility for HIV and syndromic STI testing alongside a panel of other maintenance tests. This period is based on the South African PrEP guidelines ([26](#_ENREF_26)), alongside the resource planning of the International Partnership for Microbicides (IPM), the producer of a leading dapivirine IVR (personal communication). Injectable ARVs are assumed to require monthly engagement with the system to receive new injections. We assume no shipping taxes or customs excises, and take a provider perspective as, in the South African context, the public health system is most likely to finance the rollout of new prevention technologies.

Oral PrEP is estimated to have an annual cost of $75 ($70-$130), with the unit price taken from costing studies and the Clinton Health Access Initiative ceiling price list ([49](#_ENREF_49))([43](#_ENREF_43)).The SILCS diaphragm is estimated to cost $5.19 per diaphragm, whilst gel use is estimated at 4ml per dose in an average of two sex acts per week.([29](#_ENREF_29)) Assuming 10% wastage for gels, users require seven 70ml tubes of gel per year at US$3.69 per tube, with an annual gel cost of US$25.83 (CONRAD, personal communication). Due to their continued development, unit costs for the injectable ARV are not available. One option, used in other studies have assumed similarities in costs to oral PrEP.([1](#_ENREF_1), [30](#_ENREF_30)) We explore a range of injectable costs through a sensitivity analysis, but base our mean product cost on that of the vaginal ring, however note that the higher requirements for health system utilisation of an injectable regimen will likely make this product cost more per person per year.

Cost parameters were taken from peer-reviewed sources where possible, with programmatic experts approached to inform assumptions around new products or unknown factors. All costs are presented in 2015 US Dollars (US$). Non-tradable goods (training and labour costs for example) were inflated using local currency inflation rates, whilst tradable goods (product costs) were inflated using the US$ inflation rate.

*Unwanted or mistimed pregnancies*

Where new HIV prevention products offer contraceptive properties in addition to HIV protection, secondary benefits will accrue. We calculate the additional impact of contraceptive properties among women who are not currently using modern contraceptives through estimating the number of pregnancies averted by product use. The likelihood of getting pregnant without using contraception was estimated as 40% based on DHS data for South Africa.([11](#_ENREF_11)) As in Lepine et al 2013 ([10](#_ENREF_10)), we define the added benefit of new products as the difference between this figure and the annual likelihood of conception when using a new product multiplied by the number of women using each product. For all products, we parameterise contraceptive aspects of the model using the characteristics of the SILCS diaphragm. The annual probability of pregnancy while using the SILCS diaphragm with a contraceptive gel is 17.8% for typical use and 13.7% for perfect use per year.([31](#_ENREF_31))

Table S3:1 Included costs

| Provision | 1^st^ year by month | | | | |
| --- | --- | --- | --- | --- | --- |
|  | Initiation | | Maintenance | | |
|  | 0 | 1 | 4 | 7 | 10 |
| HIV Test | x | x | x | x | x |
| Creatinine Clearance and chemistry panel | x | x | x | x | x |
| Urine pregnancy test | x |  |  |  |  |
| Syphilis test (RPR) | x |  |  |  |  |
| Syndromic STI screening | x | x | x | x | x |
| Address side effects |  | x | x | x | x |
| Adherence counselling |  | x | x | x | x |
| Product distribution | x | x | x | x | x |
| Behavioural sexual risk reduction counselling | x | x | x | x | x |

*Injectables are assumed to be delivered monthly not quarterly, though the suite of testing is only assumed to be completed quarterly as per current PrEP guidelines

Adapted from South Africa PrEP guidelines, May 2016 Final Draft

**Averted DALYs and costs**

The average age of infection was assumed to be 20 among women aged 16-24 and 30 among women aged 25-49 and FSWs and varied in a one-way sensitivity analysis. After the first 3 years of asymptomatic HIV, those with no ART access live 5 years with symptomatic HIV followed by 2 years of AIDS ending in palliative care and death ([32](#_ENREF_32)). We use estimates of life expectancy for those on ART and those uninfected from a South African cohort.([33](#_ENREF_33)) Loss-to-follow up is assumed to be zero, rendering this analysis on the conservative side, however the lower levels of use assumed in the sensitivity analysis could be interpreted as loss to follow up as the impact model used does not consider adherence over time. Standard DALY estimates are used for HIV/AIDS with and without ART taken from the 2010 Global Burden of Disease study.([14](#_ENREF_14)) Disease progression and costs averted are estimated separately for the proportion of HIV positive persons receiving ART (assumed to be 71% of HIV positive persons([12](#_ENREF_12)), though raised to 80% and 90% in a one-way sensitivity analysis), whilst lifetime treatment costs for HIV positive persons receiving ART are estimated by multiplying expected life expectancy on ART ([33](#_ENREF_33)) by annual cost estimates of Meyer-Rath et al.([12](#_ENREF_12)) and discounting. For those not accessing ART, we estimate average lifetime HIV/AIDS health care costs using inpatient cost data, varying this in a sensitivity analysis ([34](#_ENREF_34)).

As per DHS data, we assume that 92% of pregnant women access antenatal care and delivery ([11](#_ENREF_11)) with visits using 30 minutes of nurse time over four prenatal visits.([35](#_ENREF_35)) Our delivery cost estimate is a weighted average of five possible delivery outcomes: healthy child delivery, low birth-weight child, neonatal death, stillbirth and miscarriage. We follow Lepine et al. ([10](#_ENREF_10)) in estimating costs averted from the prevention of unintended pregnancies by separating unintended births, and mistimed births that occur early but would have occurred in the future. In the 2003 DHS survey, among women who did not want their last child (n = 1,434 representing 52% of total women), 49% declare that the last child was unwanted and 51% declare that they would have liked to have this birth in the future([11](#_ENREF_11)). We follow Trussell ([36](#_ENREF_36)) to assume that a mistimed birth would have occurred two years later, and incorporate a 10.5% abortion rate ([37](#_ENREF_37)) by assuming that abortion amongst wanted pregnancies is zero, whilst the abortion rate among women with mistimed or unwanted births is estimated at 20.2% (10.5/0.52).

**Sensitivity analysis**

We adhere to the ISPOR guidelines for a robust sensitivity analysis, and only apply probabilistic distributions around appropriate parameters.([38](#_ENREF_38)) The primary outcome measure used in the sensitivity analysis is product cost per person per year. At this stage we do not take into account sensitivity due to fixed costs, such as staff training or media costs.

From the literature and expert opinion, we derive a reasonable range of uncertainty around each parameter central estimate. Where more than one estimate was found in the literature, we specify upper and lower bounds according to the smallest and greatest values found and use a uniform distribution to sample random draws. Where parameters are reported in the literature with estimates of statistical uncertainty, e.g. standard errors, these are recorded and an applicable distributional form assigned. Where there is no variation in reported parameters, we use expert opinion to set higher and lower bounds for parameters, and use a uniform distribution. We run a Monte Carlo simulation with 1,000 draws for each aggregated parameter of interest (e.g. cost per person per year). Results are reported with the mean and inter-quartile range for each measure.

Separate PSAs were run on the cost and cost-effectiveness models. We incorporate variation in cost parameters in the cose-effectiveness model one-way sensitivity analysis by using the 25th and 75th percentile cost estimates, and sample a uniform distribution between these quartiles in the PSA to reflect the large degree of uncertainty in their estimation.

**Results**

The average variable cost per person per year by product is shown in table S3:2. First year costs were higher due to the more intensive initiation period. First year costs ranged from $281 for gel, adding the SILCs diaphragm only increased costs by $6. The highest estimated first year costs were for injectables ($625).

Modelling estimates were sensitive to direct product costs and the number of visits required per year. The higher frequency of visit required for monthly injections increased costs for the injectable ARV substantively, and injectable costs are very sensitive to the frequency of dosage. The remaining four products have a very similar patterns of contact with the health system, and have similar cost estimates. Microbicide gel and SILCS diaphragm (with gel) costs were very sensitive to the number of sex acts per year, as gel use is coitally specific. Fixed costs were mostly driven by assumptions made around the mass media profile for each product which will be variable depending on available resources. There was limited data to base these assumptions on, and the modelling literature has used a variety of assumptions around mass media strategies, synergies, and potential costs. Bottom up data are available for individual programmatic activities in Malawi and Zambia, however, these were not included in this model due to a) the context specific nature of the costs themselves, and b) the lack of evidence to predict total social marketing spend by population group. Instead, the standardised overhead cost applies to each population across each product.

Table S3:2: Resource use per prevention product visit – first visit and maintenance visits

|  | First two visits combined  (month 0 and month 3) | | Maintenance visits | |
| --- | --- | --- | --- | --- |
| **Product** | **Nurse time (hours)** | **Counsellor time (hours)** | **Nurse time (hours)** | **Counsellor time (hours)** |
| Oral PrEP | 0.5 | 0.5 |  | 0.5 |
| Microbicide | 0.5 | 0.5 |  | 0.5 |
| SILCS diaphragm | 0.5 | 0.5 |  | 0.5 |
| Vaginal ring | 0.5 | 0.5 |  | 0.5 |
| Injectable | 0.5 | 0.5 | 0.25 | 0.5 |

**Comparison with other studies**

These cost profiles are generally 1.5-3 times greater than other estimates in the literature. Given that the model is parameterised using similar inputs to other studies, it is likely that this increase is driven by the incorporation of full chemistry panels and syndromic STI tests as per South African guidelines – most other models do not include these costs.

**Limitations**

As with any modelling exercise, this model is a simplification of reality. To make the model operational, we make a number of assumptions around product availability and rollout. Firstly, we assume that products become available across South Africa at the same time, as indicated by assumptions around training in each health facility and mass media costs. In reality there would likely be a smaller-scale pilot phase of rollout. Depending on how roll-out occurred, it is unclear *a priori* how this would affect these estimates. Secondly, we assume that there are no economies of scale within product rollout, for example assuming that all training sessions are of the same size and are held by each provider individually. We also assume no synergies in rollout across products, for example the use of mass media to promote more than one PrEP delivery system, or the reduction in potential user reticence to use a new product in previous products have been marketed well. Both of these assumptions are likely to make our estimates conservative.

Thirdly, we assume that guidelines around PrEP are adhered to perfectly, though we allow for treatment coverage to be less than 100%, as has been observed. This is likely to reduce effectiveness of products and thus reduce averted costs; the impact of imperfect adherence is also likely to be different across the products considered. We also assume that the published guidelines for oral PrEP among female sex workers in South Africa, so far the only available PrEP guidelines, are applicable for all populations and all products – this is the cause of the similarity in resource use estimates across products. In reality, there is likely to be some variation across products in these factors, however it is unclear before their introduction and guideline publication how, for example, three-monthly HIV testing, might vary across products in reality.

**Cost model results**

Table S3:3 and Figure S3:1 detail the results of a probabilistic sensitivity analysis on the cost model, alongside the interquartile range of estimates. In this analysis, we only use the first-year cost, which results in a more conservative analysis given the anticipated reduction in costs in later years, particularly due to less intensive training.

Figure S3:1: Average variable costs per product (probabilistic approach)


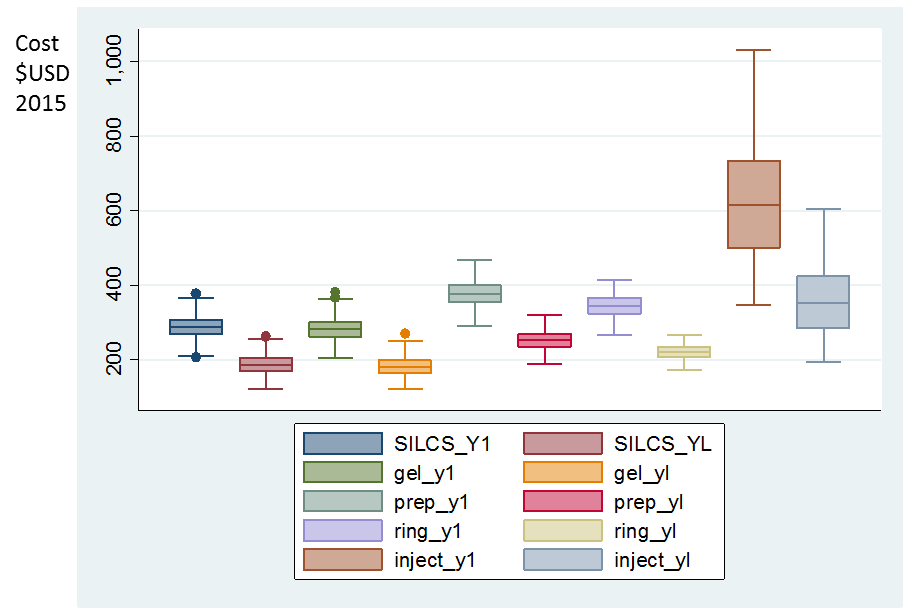


Table S3:3 Annual variable product cost per person per year, first year of introduction

| **Product** | **Point estimate** | **Lower Credible Interval/95%CI** | **Upper Credible Interval/95%CI** |
| --- | --- | --- | --- |
| Oral Prep | $289 | $269 | $309 |
| Vaginal Ring | $258 | $243 | $273 |
| Microbicide Gel | $212 | $192 | $230 |
| Injectable | $438 | $349 | $516 |
| SILCS Diaphragm and Microbicide Gel | $217 | $199 | $235 |

Table S3:4 Cost model inputs

|  | **Central value (Costs US$ 2015)** | **Upper Bound (if available)** | **Lower Bound (if available)** | **Source** | **PSA distribution (if applicable)** |
| --- | --- | --- | --- | --- | --- |
| **Fixed costs** |  |  |  |  |  |
| Number of public sector facilities | 3182 |  |  | ([39](#_ENREF_39)) |  |
| Training cost per facility | 10904 |  |  | Calculation and ([26](#_ENREF_26)) | Uniform (+- 25%) |
| Annual refresher cost per facility | 3635 |  |  | Calculation and ([26](#_ENREF_26)) | Uniform (+- 25%) |
| Mass media (total budget) | 5000000 | 5,000,000 | 1,000,000 | ([1](#_ENREF_1)) | Uniform (+- 25%) |
|  |  |  |  |  |  |
| **Wage of staff providing products** |  |  |  |  |  |
| Nurse wage per hour | 19.11 | 24 | 8 | ([40](#_ENREF_40)) | Uniform (+- 25%) |
| HIV counsellor wage per hour | 14.42 |  |  | ([41](#_ENREF_41)) | Uniform (+- 25%) |
| Physician wage per hour | 75.09 |  |  | ([15](#_ENREF_15)) | Uniform (+- 25%) |
|  |  |  |  |  |  |
| **Consumables** |  |  |  |  |  |
| HIV test | 6.19 | 6.75 | 4.67 | ([42](#_ENREF_42), [43](#_ENREF_43)) | Uniform (+- 25%) |
| Chemistry panel inc. creatinine clearance | 33.03 | 40 | 30 | ([42](#_ENREF_42)) | Uniform (+- 25%) |
|  |  |  |  |  |  |
| **Averted costs - Pregnancy** |  |  |  |  |  |
| Pregnancy outcome cost (healthy) | 75.6 |  |  | ([44](#_ENREF_44)) | Uniform (+- 50%) |
| Pregnancy outcome cost (Low birth weight) | 1570.8 |  |  | ([44](#_ENREF_44)) | Uniform (+- 50%) |
| Pregnancy outcome cost (Neonatal death) | 3724.6 |  |  | ([44](#_ENREF_44)) | Uniform (+- 50%) |
| Pregnancy outcome cost (Stillbirth) | 75.6 |  |  | ([44](#_ENREF_44)) | Uniform (+- 50%) |
| Pregnancy outcome cost (Miscarriage) | 73.1 |  |  | ([44](#_ENREF_44)) | Uniform (+- 50%) |
| Spontaneously aborted pregnancy | 36.3 |  |  | ([44](#_ENREF_44)) | Uniform (+- 50%) |
|  |  |  |  |  |  |
| **Pregnancy costs** |  |  |  |  |  |
| Average number of days of work lost due to pregnancy | 3 |  |  | Assumption |  |
| Number of prenatal visits per pregnancy | 4 |  |  | ([11](#_ENREF_11)) |  |
| Proportion of births that are really unwanted | 49% |  |  | ([11](#_ENREF_11)) |  |
| Proportions of unwanted births that are mistimed | 51% |  |  | ([11](#_ENREF_11)) |  |
| Years when mistimed births would occur | 2 |  |  | ([45](#_ENREF_45)) |  |
| Abortion rate | 10.50% |  |  | ([46](#_ENREF_46)) |  |
| Abortion rate among unintended pregnancies | 20.20% |  |  | ([11](#_ENREF_11)) |  |
| Delivery rate among unintended pregnancies | 79.80% |  |  | ([11](#_ENREF_11)) |  |
| Number of visits for abortion (pre-act) | 3 |  |  | ([47](#_ENREF_47)) |  |
| % of women receiving antenatal care | 92% |  |  | ([11](#_ENREF_11)) |  |
| % of women where delivery was assisted by skilled health worker | 92% |  |  | ([11](#_ENREF_11)) |  |
| Number of prenatal visits needed when pregnant | 4 |  |  | ([11](#_ENREF_11)) |  |
|  |  |  |  |  |  |
|  |  |  |  |  |  |
| **Product-specific costs** |  |  |  |  |  |
| SILCS Diaphragm cost per year | 5.19 | 6 | 4 | Kessel (Personal communication) {Kessel, 2015 #1464} | Uniform (bounds) |
| Oral PrEP drug cost per year | 75 | 130 | 70 | Assumed using ranges from primary studies:  CHAI ceiling price 2015: $67.20/year ([49](#_ENREF_49))  South African Investment case: $75.13([43](#_ENREF_43));  CHAI ceiling price 2010 $75 cited in ([50](#_ENREF_50))Walenksy([42](#_ENREF_42)) (direct link no longer available). ([1](#_ENREF_1)) | Uniform (bounds) |
| Microbicide gel tube cost | 3.69 | 4.5 | 3 | Kessel (Personal communication) {Kessel, 2015 #1464}, Population council (personal communication) | Uniform (bounds) |
| Intravaginal ring cost | 6 | 7 | 5 | Bodarky, (personal communication){Bodarky, 2015 #1465} | Uniform (bounds) |
| Injectable cost per injection | 5 | 7 | 5 | Assumption from cost of IPM IVR | Uniform (bounds) |
| Number of sex acts per year | 104 | 208 | 20 | ([29](#_ENREF_29)) | Uniform (bounds) |
| Quantity of gel used per sex (in ml) | 4 |  |  | Kessel (Personal communication) {Kessel, 2015 #1464} | Uniform (bounds) |
|  |  |  |  |  |  |
| **Clinic visits per year by product** |  |  |  |  |  |
| SILCS diaphragm, microbicide gel, oral PrEP, IVR | 4 | 4 | 3 | ([26](#_ENREF_26)) |  |
| Injectable ARV | 12 | 12 | 4 | ([51](#_ENREF_51)) |  |
|  |  |  |  |  |  |
| Discount rate | 0.03 |  |  | ([52](#_ENREF_52)) |  |

**Supplementary file S4: Division of fixed costs across populations**

To estimate population-level cost-effectiveness, we account for the allocation of programme fixed costs across the three groups differently, depending on the type of cost. We allocate facility-level fixed costs for training across general population groups according to the proportion of general population females in each group. FSW training costs are calculated through multiplying the number of FSW-specific facilities operating in South Africa by estimated training costs per facility. Mass media costs are only allocated to general population groups, proportional to size. Contraceptive research and development costs are allocated across all three groups proportional to the number of users in each. Finally, FSW peer-educator and outreach costs are estimated by assuming 12 peer-educators per site working one day per week. Table S3:1 displays these costs as allocated.

**Table S4:1 Summary of annual fixed costs by population group**

| **Assumptions** | **Training** | **Mass media** | **Contraceptive R+D** | **Peer educator / outreach costs** | **Total** |
| --- | --- | --- | --- | --- | --- |
| Women 15-24 | $12,872,686.84 | $1,855,023.45 | $1,833,391.29 |  | $16,561,101.57 |
| Women 25-49 | $21,824,143.70 | $3,144,976.55 | $3,108,301.74 |  | $28,077,422.00 |
| FSWs | $1,112,217.70 | $- | $58,306.98 | $403,920.00 | $1,574,444.67 |

**Supplementary file S5: HIV infections averted**

**Table S5:1 Total HIV infections averted by scenario**

| **Women 15-24** | **HIV infections** |  |  | **HIV Infections averted** | |  |
| --- | --- | --- | --- | --- | --- | --- |
| **Scenario** | **Low incidence** | **Central Incidence** | **High incidence** | **Low incidence** | **Central Incidence** | **High incidence** |
| Counterfactual | *69,052* | *108,266* | *213,123* |  |  |  |
| Scenario 1: Oral PrEP with HIV protection only | 65,020 | 101,945 | 200,680 | 4,031 | 6,321 | 12,443 |
| Scenario 2: Oral PrEP & vaginal ring with HIV protection only | 64,238 | 100,719 | 198,265 | 4,814 | 7,548 | 14,858 |
| Scenario 3: MPT ring plus oral PrEP | 62,234 | 97,577 | 192,080 | 6,818 | 10,690 | 21,043 |
| Scenario 4: All five products with HIV protection only | 63,911 | 100,206 | 197,256 | 5,141 | 8,060 | 15,867 |
| Scenario 5: All five products with multipurpose protection | 51,946 | 81,446 | 160,327 | 17,106 | 26,820 | 52,796 |
|  |  |  |  |  |  |  |
| **Women 25+** |  | **HIV infections** |  |  | **HIV Infections averted** |  |
| **Scenario** | **Low incidence** | **Central Incidence** | **High incidence** | **Low incidence** | **Central Incidence** | **High incidence** |
| Counterfactual | *69,100* | *93,285* | *201,543* |  |  |  |
| Scenario 1: Oral PrEP with HIV protection only | 58,675 | 79,212 | 171,137 | 10,425 | 14,074 | 30,406 |
| Scenario 2: Oral PrEP & vaginal ring with HIV protection only | 56,993 | 76,940 | 166,229 | 12,108 | 16,345 | 35,314 |
| Scenario 3: MPT ring plus oral PrEP | 56,747 | 76,608 | 165,511 | 12,354 | 16,677 | 36,031 |
| Scenario 4: All five products with HIV protection only | 56,310 | 76,019 | 164,238 | 12,790 | 17,267 | 37,304 |
| Scenario 5: All five products with multipurpose protection | 51,915 | 70,085 | 151,418 | 17,186 | 23,201 | 50,125 |
|  |  |  |  |  |  |  |
| **FSW** |  | **HIV infections** |  |  | **HIV Infections averted** |  |
| **Scenario** | **Low incidence** | **Central Incidence** | **High incidence** | **Low incidence** | **Central Incidence** | **High incidence** |
| Counterfactual | *1,026* | *2,020* | *3,231* |  |  |  |
| Scenario 1: Oral PrEP with HIV protection only | 877 | 1,727 | 2,763 | 149 | 293 | 469 |
| Scenario 2: Oral PrEP & vaginal ring with HIV protection only | 737 | 1,451 | 2,321 | 289 | 569 | 910 |
| Scenario 3: MPT ring plus oral PrEP | 739 | 1,455 | 2,329 | 287 | 564 | 903 |
| Scenario 4: All five products with HIV protection only | 747 | 1,471 | 2,353 | 279 | 549 | 878 |
| Scenario 5: All five products with multipurpose protection | 685 | 1,349 | 2,158 | 341 | 671 | 1,073 |

**Supplementary file S6: One-way sensitivity analyses**

**Scenario 2.1: Oral PrEP and MPT ring**


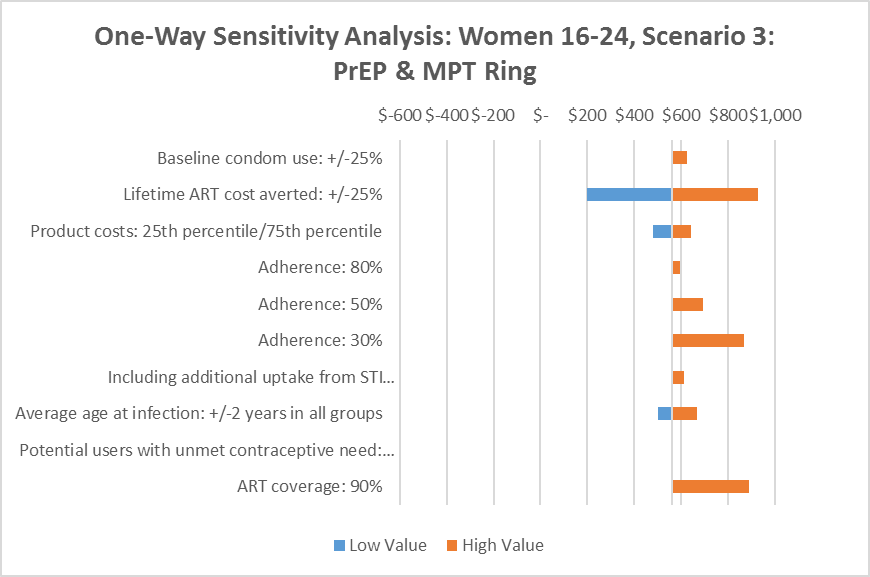


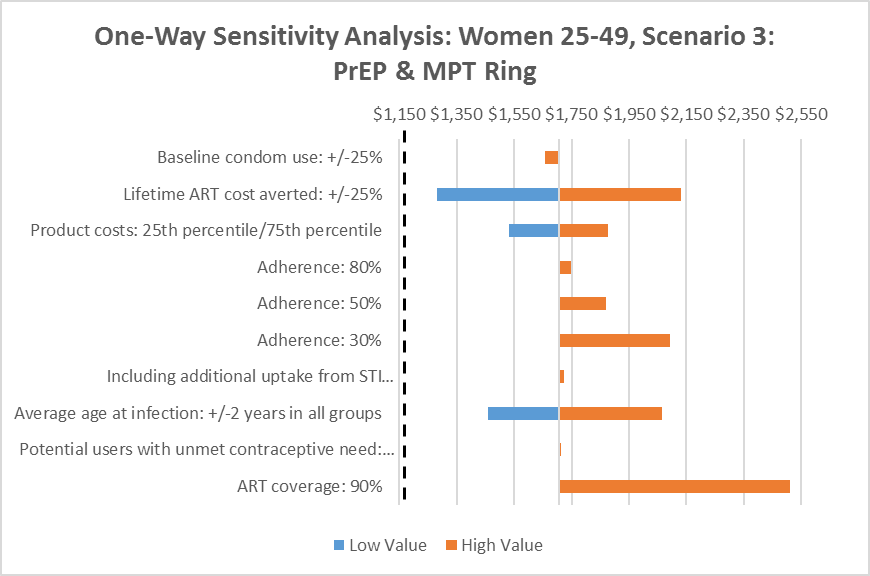


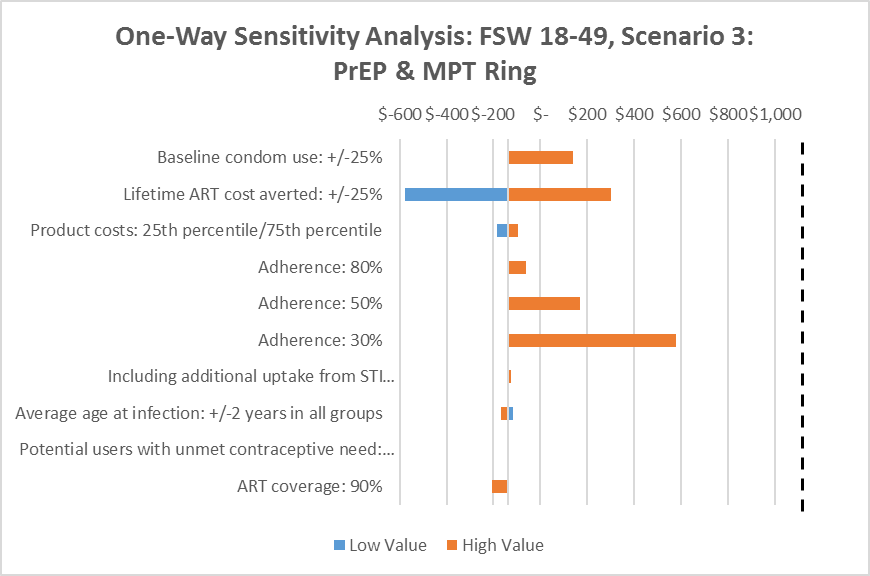


**Supplementary file S7: Net intervention costs**

Figure S7:1: Net intervention costs by population


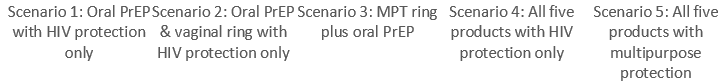


**Supplementary file S8: Additional data from probabilistic sensitivity analysis**

Below are a series of cost-effectiveness plane plots showing the results of 1000 Monte Carlo simulations carried out for the probabilistic sensitivity analysis. All ICERs are plotted as coloured shapes. Results are plotted on the cost-effectiveness plane and, similarly to Figure 5, when compared to the dashed WTP threshold line, can be interpreted as cost-effective when they lie to the south east. These simulations were also used to generate the CEAC curves in the main analysis.

# Supplementary material reference list

1. Smith JA, Anderson S-J, Harris KL, McGillen JB, Lee E, Garnett GP, et al. Maximising HIV prevention by balancing the opportunities of today with the promises of tomorrow: a modelling study. The Lancet HIV. 2016;3(7):e289-e96.

2. World Bank. Population Estimates and Projections 2014 [01/08/2014]. Available from: <http://data.worldbank.org/data-catalog/population-projection-tables>.

3. South African National AIDS Council. Estimating the Size of the sex worker population in South Africa, 2013. 2013.

4. Shisana O, Rehle T, Simbayi L, Zuma K, Jooste S, Zungu N, et al. South African National HIV Prevalence, Incidence and Behaviour Survey 2012. Cape Town: HSRC Press, 2014.

5. Zuma K, Shisana O, Rehle TM, Simbayi LC, Jooste S, Zungu N, et al. New insights into HIV epidemic in South Africa: key findings from the National HIV Prevalence, Incidence and Behaviour Survey, 2012. Afr J AIDS Res. 2016;15(1):67-75.

6. Baeten JM, Palanee-Phillips T, Brown ER, Schwartz K, Soto-Torres LE, Govender V, et al. Use of a vaginal ring containing dapivirine for HIV-1 prevention in women. New England Journal of Medicine. 2016.

7. Van Damme L, Corneli A, Ahmed K, Agot K, Lombaard J, Kapiga S, editors. The FEM-PrEP trial of emtricitabine/tenofovir disoproxil fumarate (Truvada) among African women. 19th Conference on retroviruses and opportunistic infections; 2012.

8. Marrazzo JM, Ramjee G, Richardson BA, Gomez K, Mgodi N, Nair G, et al. Tenofovir-Based Preexposure Prophylaxis for HIV Infection among African Women. New England Journal of Medicine. 2015;372(6):509-18.

9. Winner B, Peipert JF, Zhao Q, Buckel C, Madden T, Allsworth JE, et al. Effectiveness of Long-Acting Reversible Contraception. New England Journal of Medicine. 2012;366(21):1998-2007.

10. Lépine A, Nundy N, Kilbourne-Brook M, Siapka M, Terris-Prestholt F. Cost-Effectiveness of Introducing the SILCS Diaphragm in South Africa. PLoS ONE. 2015;10(8):e0134510.

11. South Africa Department of Health MRC. South Africa Demographic and Health Survey 2003. Pretoria: South Africa Deparment of Health, 2007.

12. Meyer-Rath G. National ART Cost Model, South Africa. Health Economics and Epidemiology Research Office, Boston University/ University of the Witwatersrand, Johannesburg., 2016.

13. World Health Organisation. National Burden of Disease Studies: A Practical Guide 2.0. 2001.

14. Vos T, Barber RM, Bell B, Bertozzi-Villa A, Biryukov S, Bolliger I, et al. Global, regional, and national incidence, prevalence, and years lived with disability for 301 acute and chronic diseases and injuries in 188 countries, 1990–2013: a systematic analysis for the Global Burden of Disease Study 2013. The Lancet. 2015;386(9995):743-800.

15. Meyer-Rath G, Schnippel K, Long L, MacLeod W, Sanne I, Stevens W, et al. The Impact and Cost of Scaling up GeneXpert MTB/RIF in South Africa. PLoS ONE. 2012;7(5):e36966.

16. Hanscom B, Janes HE, Guarino PD, Huang Y, Brown ER, Chen YQ, et al. Preventing HIV-1 Infection in Women using Oral Pre-Exposure Prophylaxis: A Meta-analysis of Current Evidence. J Acquir Immune Defic Syndr. 2016.

17. AVAC. HIV Prevention Research and Develoment Database: AVAC; 2015 [2/11/2016]. Available from: <http://www.avac.org/pxrd>.

18. STATS SA. South African Census 2011. 2011.

19. Johnston LG, Sabin K. Sampling Hard-to-Reach Populations with Respondent Driven Sampling. Methodological Innovations Online. 2010;5(2):38-48.

20. Volz E, Wejnert C, Deganii I, Heckathorn D. Respondent-Driven Sampling Analysis Tool (RDSAT) 6.0. New York: Cornell University; 2007.

21. Terris-Prestholt F, Quaife M, Vickerman P. Parameterising User Uptake in Economic Evaluations: The role of discrete choice experiments. Health Economics. 2016;25:116-23.

22. McFadden D. Conditional logit analysis of qualitative choice behaviour. In: Zarembka P, editor. Frontiers in econometrics. New York: Academic Press; 1974.

23. Henscher D, Rose J, Greene W. Applied Choice Analysis: A Primer. Cambridge: Cambridge University Press; 2005.

24. Philips H, Mahr D, Remmen R, Weverbergh M, De Graeve D, Van Royen P. Predicting the place of out-of-hours care-A market simulation based on discrete choice analysis Ireland: Elsevier Ireland Ltd (P.O. Box 85, Limerick, Ireland); 2012 [cited 106 (Philips, Remmen, Van Royen) University of Antwerp, Department of Family Medicine, Centre for General Practice, Universiteitsplein 1, B-2610 Antwerp, Belgium]. 3:[284-90]. Available from: <http://ovidsp.ovid.com/ovidweb.cgi?T=JS&PAGE=reference&D=emed10&NEWS=N&AN=2012407174>.

25. Quaife M, Eakle R, Cabrera M, Vickerman P, Tsepe M, Cianci F, et al. Preferences for ARV-based HIV prevention methods among men and women, adolescent girls and female sex workers in Gauteng Province, South Africa: a protocol for a discrete choice experiment. BMJ Open. 2016;6(6):e010682.

26. Department of Health South Africa. Guidelines for Expanding Combination Prevention and Treatment Options for Sex Workers: Oral Pre-Exposure Prophylaxis (PrEP) and Test and Treat (T&T). Final Draft 11 May 2016. 2016.

27. Eaton JW, Menzies NA, Stover J, Cambiano V, Chindelevitch L, Cori A, et al. Health benefits, costs, and cost-effectiveness of earlier eligibility for adult antiretroviral therapy and expanded treatment coverage: a combined analysis of 12 mathematical models. The lancet global health. 2013;2(1):23-34.

28. Chola L, McGee S, Tugendhaft A, Buchmann E, Hofman K. Scaling Up Family Planning to Reduce Maternal and Child Mortality: The Potential Costs and Benefits of Modern Contraceptive Use in South Africa. PLoS ONE. 2015;10(6):e0130077.

29. Smith J, Nyamukapa C, Gregson S, Lewis J, Magutshwa S, Schumacher C, et al. The Distribution of Sex Acts and Condom Use within Partnerships in a Rural Sub-Saharan African Population. PLoS ONE. 2014;9(2):e88378.

30. Glaubius RL, Hood G, Penrose KJ, Parikh UM, Mellors JW, Bendavid E, et al. Cost-effectiveness of Injectable Preexposure Prophylaxis for HIV Prevention in South Africa. Clin Infect Dis. 2016;63(4):539-47.

31. CONRAD, editor 48th Annual Meeting of the Association of Reproductive Health Professionals2011; Las Vegas.

32. Terris-Prestholt F, Foss AM, Cox AP, Heise L, Meyer-Rath G, Delany-Moretlwe S, et al. Cost-effectiveness of tenofovir gel in urban South Africa: model projections of HIV impact and threshold product prices. BMC Infect Dis. 2014;14:14.

33. Johnson LF, Mossong J, Dorrington RE, Schomaker M, Hoffmann CJ, Keiser O, et al. Life Expectancies of South African Adults Starting Antiretroviral Treatment: Collaborative Analysis of Cohort Studies. PLoS Med. 2013;10(4):e1001418.

34. Meyer-Rath G, Brennan AT, Fox MP, Modisenyane T, Tshabangu N, Mohapi L, et al. Rates and cost of hospitalisation before and after initiation of antiretroviral therapy in urban and rural settings in South Africa. Journal of acquired immune deficiency syndromes (1999). 2013;62(3):322.

35. Piper A. Early Effects of Environment: A Comparison of Prenatal Care in South Africa and the United States: Carnegie Mellon University;; 2010.

36. Trussell J. Overstating the cost savings from contraceptive use. The European Journal of Contraception & Reproductive Health Care. 2008;13(3):219-21.

37. Robert J. South Africa: Abortion rates by province, 1997-2010. 2011.

38. Briggs AH, Weinstein MC, Fenwick EA, Karnon J, Sculpher MJ, Paltiel AD, et al. Model parameter estimation and uncertainty: a report of the ISPOR-SMDM Modeling Good Research Practices Task Force-6. Value in Health. 2012;15(6):835-42.

39. Health Systems Trust. Health Indicators 2015 [9/9/2016].

40. Larson B, Schnippel K, Ndibongo B, Long L, Fox MP, Rosen S. How to estimate the cost of point-of-care CD4 testing in program settings: an example using the Alere Pima Analyzer in South Africa. PLoS One. 2012;7(4):e35444.

41. Bill and Melinda Gates Foundation. PrEP Bottom up cost model. Seattle: BMGF, 2011.

42. Walensky RP, Park JE, Wood R, Freedberg KA, Scott CA, Bekker LG, et al. The cost-effectiveness of pre-exposure prophylaxis for HIV infection in South African women. Clin Infect Dis. 2012;54(10):1504-13.

43. Department of Health South Africa, South African National AIDS Council. South African HIV and TB Investment Case - Summary Report Phase 1. Pretoria: 2016.

44. Owusu-Edusei K, Jr., Gift TL, Ballard RC. Cost-effectiveness of a dual non-treponemal/treponemal syphilis point-of-care test to prevent adverse pregnancy outcomes in sub-Saharan Africa. Sex Transm Dis. 2011;38(11):997-1003.

45. World Health Organization Department of Reproductive Health and Research Family Planning: A Global Handbook for Providers (2011 update). 2011.

46. World Bank. World Bank Development Indicators 2012. 2012.

47. Grossman D, Constant D, Lince N, Alblas M, Blanchard K, Harries J. Surgical and medical second trimester abortion in South Africa: A cross-sectional study. BMC Health Services Research. 2011;11(1):1-9.

48. Clinton Health Access Initiative. Antiretroviral Ceiling Price List. 2014.

49. Clinton Health Access Initiative. 2015 ANTIRETROVIRAL (ARV) CHAI REFERENCE PRICE LIST 2015 [23/11/2017]. Available from: <https://clintonhealthaccess.org/content/uploads/2016/01/2015-CHAI-ARV-Reference-Price-List.pdf>.

50. Price JT, Wheeler SB, Stranix-Chibanda L, Hosek SG, Watts DH, Siberry GK, et al. Cost-Effectiveness of Pre-exposure HIV Prophylaxis During Pregnancy and Breastfeeding in Sub-Saharan Africa. JAIDS Journal of Acquired Immune Deficiency Syndromes. 2016;72:S145-S53.

51. AVAC. Introduction to Long-Acting Injectables. 2014.

52. Edejer T-T, Baltussen R, Adam T, Hutubessy R, Acharya A, Evans D, et al. WHO guide to cost-effectiveness analysis. Geneva: World Health Organization. 2003.
